# Fluid Management in acute cardiac condition and superimposed COVID-19 infection. Do we need a careful revision?

**DOI:** 10.22088/cjim.12.3.356

**Published:** 2021-04

**Authors:** Mohammadreza Naderian, Ali Sheikhy, Seyyed Mojtaba Ghorashi, Masih Tajdini, Saeed Sadeghian, Kaveh Hosseini

**Affiliations:** 1 **Cardiovascular Research Institute, Tehran Heart Center, Tehran University of Medical Sciences, Tehran, Iran**

**Keywords:** COVID-19, Fluid management, Cardiac disease

**Dear Sir,**

Cardiac diseases are one of the most important causes of death and complications among patients with COVID-19 infection. This study was approved by the Ethics Committee of Tehran University of Medical Sciences (IR.TUMS.VCR.REC.1399.011). From 5 June 2020 to 4 July 2020, 190 patients with COVID-19 infection and cardiovascular diseases were admitted to Tehran Heart Center (1) (a tertiary center for the treatment of heart diseases in the central zone of Tehran, the capital of Iran); among them, 55 patients had both acute cardiac condition (STEMI, NSTEMI, and DHF) and positive rRT-PCR (by GeneoDx kit GZ-TRM2 China) or chest CT in favor of COVID-19 infection. Like other studies mentioned in the previous articles, differentiating between pulmonary edema and COVID-19 infection is a challenge, especially in the absence of widespread use of accurate diagnostic tests.

We compared these groups of patients in our database; 20 patients with prerenal azotemia – defined as the ratio of blood urea nitrogen to creatinine of more than 20, after exclusion of secondary causes - at the time of admission and 30 patients without prerenal azotemia (table 1).

 Five patients were excluded from the study because they received hemodialysis as renal replacement therapy before admission, and there were no accurate measures for detecting prerenal azotemia.

Regarding the background prerenal azotemia, they had not received enough fluids in the first 24 hours. The prerenal azotemia group eventually experienced more death (50% compared with 13.3%) and more death composites, acute renal failure, and intubation (60% compared with 26.5%). Further investigation of our data revealed that the amount of fluid administration was inversely associated with hospitalization length (Spearman’s rho correlation coefficient: - 0.31, p0.06). There were no significant differences between the amount of serum therapy in the dead and survived patients, intubated and non-intubated patients, and ARDS and non-ARDS patients. 

This study showed that high BUN to creatinine ratio was associated with poor outcomes at the time of admission. Patients with concurrent COVID-19 infection and acute cardiac conditions are at risk of dehydration, prerenal azotemia, and acute renal failure. Renal dysfunction will result in oliguria and electrolyte imbalance, leading to a vicious cycle of the cardio-renal syndrome that ultimately causes death.

 Radiological presentations of COVID-19 infection (such as ground-glass opacification and pleural effusion) overlap with the features of patients with heart failure. In such a situation, cardiologists have to start diuretics or at least hesitate to administer the appropriate amount of fluid. Besides, fever, oral fluid intolerance, vomiting, and increased insensible water loss via respiration after COVID-19 infection make the patient more vulnerable to dehydration and renal dysfunction. Although a recent meta-analysis has proposed that the COVID-19 virus does not directly affect renal function; i.e., kidney injuries in the course of COVID-19 infection are mainly the results of dehydration, hypoxemia, and subsequent acute kidney injury (2), it appears that renal dysfunction during the course of COVID-19 infection regardless of underlying origins is connected with poor outcome (3). 

In concordance with previous expert opinions (4), our study emphasizes the use of balanced fluid therapy in patients with background cardiac comorbidities. It is worth mentioning that even though some researchers recommend conservative fluid administration for patients with COVID-19 infection and especially those with ARDS (5), our findings imply the importance of both respiratory care and proper fluid management, which prevents further kidney injury and down-stream medical complications. On the other hand, invasive hemodynamic monitoring and fluid management of these particular groups of patients could be considered, and the authors suggest the use of invasive hemodynamic monitoring in all patients with COVID-19 and active cardiac patients for better fluid and hemodynamic management.

**Table 1:** Demographic and clinical information of patients

	**Patients with prerenal azotemia at admission** **(N = 20)**	**Patients without prerenal azotemia at admission** **(N = 30)**	***P Value***
**Before admission**			
Age, year, mean ± SD	70.50 ± 10.18	64 ± 13.95	0.17
Men, n (%)	7 (35%)	25 (83%)	***<0.01***
Previous history of DM, n (%)	14 (73.7%)	15 (51.7%)	0.12
Previous history of HTN, n (%)	13 (68.4%)	17 (58.6%)	0.49
Previous history of DLP, n (%)	8 (42.1%)	13 (44.8%)	0.85
Previous history of IHD, n (%)	5 (26.3%)	12 (40%)	0.32
Previous history of MI, n (%)	4 (21%)	10 (34.5%)	0.31
Previous history of HF, n (%)	5 (26.3%)	2 (6.9%)	0.06
Current cigarette smoking, n (%)	2 (10.5%)	13 (44.8%)	***0.01***
Previous use of ACEI or ARB, n (%)	11 (55%)	20 (66.7%)	0.40
Previous use of diuretic, n (%)	7 (38.9%)	3 (10.3%)	0.02
Previous use of NSAIDs, n (%)	0 (0%)	1 (3.3%)	-
**At the time of admission**			
SBP, mmHg, mean ± SD	121.87 ± 21.68	128.30 ± 24.07	0.59
DBP, mmHg, mean ± SD	69.27 ± 10.81	78.56 ± 14.61	0.33
Oxygen saturation, percent, mean ± SD	88.94 ± 10.70	92.83 ± 5.20	***0.01***
Temperature, Celsius, mean ± SD	37.21 ± 0.95	36.69 ± 0.69	0.27
WBC count, per μL of blood, mean ± SD	12852 ± 5652	9928 ± 3077	***<0.01***
Lymphocyte count, per μL of blood, mean ± SD	2686 ± 3437	1802 ± 727	0.06
Blood urea nitrogen, mg/dL, mean ± SD	34.34 ± 17.84	18.00 ± 8.74	***<0.01***
Serum creatinine, mg/dL, mean ± SD	1.39 ± 0.66	1.33 ± 0.55	0.13
CRP, mg/dL, mean ± SD	7.34 ± 7.62	8.49 ± 10.33	0.30
Natural logarithm of serum troponin, ng/L, mean ± SD	6.31 ± 2.01	5.32 ± 2.85	0.41
LVEF, percent, mean ± SD	38.32 ± 10.78	42.68 ± 10.32	0.99
Received IV fluid volume in first 24h, mL, mean ± SD	587.14 ± 520.37	454.29 ± 621.29	0.30
Diuretic therapy in first 24h, n (%)	6 (42.9%)	5 (25%)	0.27
**During admission**			
ARDS, n (%)	1 (5%)	2 (6.7%)	0.80
CPR, n (%)	6 (30%)	3 10%)	0.07
Intensive unit admission, n (%)	20 (100%)	28 (93.3%)	0.23
Intubation, n (%)	9 (45%)	8 (26.7%)	0.18
**Patients’ Outcomes**			
WBC count at discharge, per μL of blood, mean ± SD	15416 ± 10037	8417 ± 3344	***<0.01***
Lymphocyte count at discharge, per μL of blood, mean ± SD	4094 ± 6459	1982 ± 884	***0.01***
Blood urea nitrogen at discharge, mg/dL, mean ± SD	103.51 ± 75.99	75.63 ± 135.63	0.90
Serum creatinine at discharge, mg/dL, mean ± SD	1.51 ± 0.87	1.97 ± 1.7	0.10
CRP level at discharge, mg/dL, mean ± SD	11.32 ± 12.29	5.92 ± 8.5	0.07
Length of hospital stay, day, median (25^th^ percentile – 75^th^ percentile)	7 (5-13)	7 (5-11)	0.96
Death, n (%)	10 (50%)	4 (13.3%)	***<0.01***
Composite of death, acute renal failure and intubation, n (%)	12 (60%)	9 (26.5%)	***0.01***

* Abbreviation: ACEI: Angiotensin Converting Enzyme inhibitor; ADRS: Acute Respiratory Distress Syndrome; ARB: Angiotensin Receptor Blocking; CKD: Chronic Kidney Disease; CPR: Cardiopulmonary Resuscitation; CRP: C-reactive Protein; dL: Deciliter, DLP: Dyslipidemia; DM: Diabetes Mellitus; EF: Ejection Fraction; HF: Heart Failure; HTN: Hypertension; IHD: Ischemic heart Disease; IQR: Interquartile Range; IV: Intravenous; L: Liter, LV: Left Ventricle; mg: milligram; MI: Myocardial Infarction; m:: Milliliter, mmHg: millimeter of mercury; ng: Nano-gram, NSAIDs: Non-steroidal Anti-inflammatory Drugs; SD: Standard Deviation, μL: microliter.
